# Plumbagin restrains hepatocellular carcinoma angiogenesis by suppressing the migration and invasion of tumor-derived vascular endothelial cells

**DOI:** 10.18632/oncotarget.14774

**Published:** 2017-01-20

**Authors:** YanFei Wei, Qi Yang, Yuan Zhang, TieJian Zhao, XueMei Liu, Jing Zhong, Jing Ma, YongXin Chen, Chuan Zhao, JunXuan Li

**Affiliations:** ^1^ Department of Physiology, Faculty of Basic Medicine, Guangxi University of Chinese Medicine, Nanning, Guangxi, 530200, China; ^2^ Department of Emergency, Tianjin Fifth Central Hospital, Binhai New Area, Tianjin 300450, China; ^3^ Department of State Key Laboratory of Medicinal Chemical Biology and College of Pharmacy, Nankai University, Tianjin, 710032, China

**Keywords:** plumbagin, HCC, angiogenesis, vascular endothelial cells, PI3K/AKT

## Abstract

Tumor occurrence and development are very complicated processes. In addition to the roles of exogenous carcinogenic factors, the body's internal factors also play important roles. These factors include the host response to the tumor and the tumor effect on the host. In particular, the proliferation, migration and activation of endothelial cells are involved in tumor angiogenesis. Angiogenesis is one of the hallmarks of cancer. In this study, we investigate whether plumbagin can abrogate angiogenesis-mediated tumor growth in hepatocellular carcinoma (HCC) and, if so, through which molecular mechanisms. We observed that in co-cultures of the human endothelial cell line EA.hy926 and the human hepatoma cell line SMMC-7721 and Hep3B, the hepatoma cells induced migration, invasion, tube formation and viability of the EA.hy926 cells *in vitro*, and these processes were inhibited by plumbagin. Real-Time PCR, Western Blot and Immunofluorescence staining showed that plumbagin treatment suppressed expression of angiogenesis pathways (PI3K-Akt, VEGF/KDR and Angiopoietins/Tie2) and angiogenic factors (VEGF, CTGF, ET-1, bFGF),which is associated with tumor angiogenesis in cancer cells and xenograft tumor tissues. Furthermore, plumbagin was also found to significantly reduce tumor growth in an orthotopic HCC mouse model and to inhibit tumor-induced angiogenesis in HCC patient xenografts. Taken together, our findings strongly suggest that plumbagin might be a promising anti-angiogenic drug with significant antitumor activity in HCC.

## INTRODUCTION

Angiogenesis is essential for solid tumors metastasis formation and growth [1]. experimental and clinical data have proved that angiogenic factors vascular endothelial growth factor (VEGF), basic fibroblast growth factor (bFGF), connective tissue growth factor (CTGF), endothelia (ET-1) and angiopoietins(Ang1/Ang2) stimulates normal vessel excessive growth contribute to cancer progression [2]. cancer cells stimulate angiogenesis in an effort to maintain and allow tumor progression [3]. Meanwhile,cancer cells by secreting growth factors connect with endothelial cells [4]. Besides, growth factor bind to receptors on endothelial cells to initiate basement membrane degradation.as the result, endothelial cell proliferation and migration, capillary tubule formation and an increase in vessel permeability [5]. In fact, endothelial cells as a tip cells contact each other creates a network and from embryo to adult remain high perception to angiogenic signals [6]. Specifically, endothelial cells were activated by pro-angiogenic factors, receptor tyrosine kinases (PI3K/Akt and RAF/MEK/ERK) signaling pathways [7]. Therefore, endothelial cells are considered to be ideal therapeutic targets resistant to anti-angiogenic therapy. Our study was designed to determine whether plumbagin regulates the migration and invasion of endothelial cells, thus inhibiting the tumor vasculature.

Plumbagin (5-hydroxy-2-methyl-1, 4-napthoquinone, PL) is a nature napthoquinone compound that is isolated from plants including Plumbago europaea and plumbago rose.Reports have shown that PL exerts potential health benefits, including antioxidant, anti-inflammatory, antibacterial, antifungal and anticancer properties [8]. PL is effective against various types of cancers, especially cancer of the hepatocellular carcinoma. Previous study demonstrated that PL significantly decreased HepG2 cell viability in a dose-dependent manner. at the same time, HepG2 cell migration and invasion was inhibited by down-regulation of MMP-2 and uPA [9]. Additionally, PL increased the Bax/Bcl-2 ratio and caspase-3/7 activity [10]. In an earlier study, plumbagin was found to be cytotoxic against the HEPA-3B hepatoma cell line [11]. Parimala, R’ investigations indicated PL against hepatoma studied in rats by 3MeDAB induced hepatoma [12]. In addition, PL not only inhibited endothelial cell proliferation, migration and tube formation but also anti-angiogenic effectively inhibits VEGF-A and CD31 expression and disrupts growth of ovarian cancer cells [13]. However, it remains unclear whether the PL inhibited tumor-derived vascular endothelial cells migration, invasion effects against angiogenesis. In this study, we have demonstrated that PL could suppress tumor-derived angiogenesis both *in vitro* and *in vivo*. More importantly, we have found that PL could indeed attenuate endothelial cell migration, invasion, tube formation and intratumoral microvessel density (MVD) through the abrogation of angiogenic factors mediated PI3K/AKT signaling pathway.

## RESULTS

### Effect of plumbagin on the migration and invasion of EAhy926 cells induced by co-culture with SMMC-7721 cells or Hep3B cells

**Figure 1:**
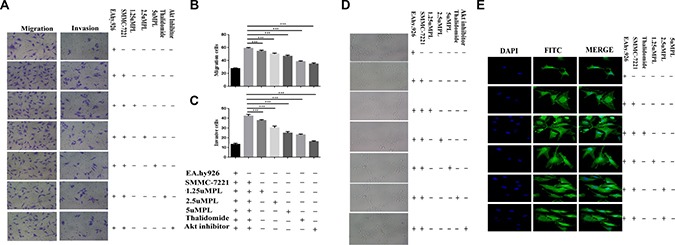
Plumbagin reduced the migration and invasion of the human endothelial cell line EA.hy926 that was induced by the human hepatoma cell line Hep3B cells (**A**) Plumbagin-depleted cells (24 h) were loaded for transwell migration (left) and Matrigel invasion assays (right). (**B**–**C**) Migration or invasion were assessed at 24 h. Fields were counted for each well. The Hep3B cells were treated with plumbagin as indicated and migration or invasion experiments were performed as in (B–C). (**D**) The co-cultured hy926 cells can also spontaneously form capillary-like structures on Matrigel, and we therefore studied the effects of plumbagin on the angiogenesis in hy926 cells. Our data showed that the number and the continuity of the capillary-like structures of the hy926 cells were all dramatically inhibited by 1.25–5 μM plumbagin in a dose-dependent manner, which suggested that plumbagin inhibited the formation of tubes that was induced by the hy926 cells *in vitro*. (**E**) The FITC–phalloidin staining assay was performed to examine the structure of F-actin. Plumbagin (5 μM) suppressed the changes in cell morphology and actin remodeling induced by the co-culture with hy926 cells. Thalidomide (25 μM) was used as the positive control, GSK690693 (10 μM) was used as the Akt inhibitor group. The data represent the mean values of three experiments ± SE.*P < 0.05, **P < 0.01, ***p < 0.001 compared to co-culture with hy926 cells.

In our study, the MTT assays revealed that treatment of various concentrations (0, 1.25 2.5, or 5 μM) of plumbagin caused no significant cytotoxicity to the EA.hy926 cells. This concentration applied to all subsequent experiments. Endothelial cell migration is one of the most important and early events during the process of angiogenesis [14]. To assess the *in vitro* anti-angiogenic property of plumbagin, we examined its effects on the chemotactic motility of endothelial cells using transwell migration and invasion assays. When EA.hy926 cells were co-cultured with SMMC-7721 cells or Hep3B cells, the migration of the cells treated with plumbagin was much less than that of the control co-culture group (co-culture of Hep3B cells and EA.hy926 cells, co-culture of SMMC-7721 cells and EA.hy926 cells) (Figure 1A–1B, Supplementary Figure 1A–1B). Similar results were obtained when EA.hy926 cells treated with plumbagin were allowed to invade the matrigel-coated polycarbonate membranes (Figure 1A–1C and Supplementary Figure 1A–1C).

### Effect of plumbagin on the capillary-like structure formation and cell morphology including F-actin remodeling induced by co-culture of EA.hy926 cells with SMMC-7721 cells or Hep3B cells

when EA.hy926 cells were cultured on matrigel three-dimensional capillary-like tubular structures formed. tube formation represents that of angiogenesis. We therefore studied the effects of plumbagin on tubulogenesis in EA.hy926 cells. Our results indicated that EA.hy926 cells can form robust tubule-like structures when seeded on growth factor–reduced two-dimensional matrigel when they are co-cultured with SMMC-7721 cells or Hep3B cells. However, treatment with plumbagin leaded to a significant dose-dependent reduction in the number and the continuity of the EA.hy926 cell capillary-like structures (Figure 1D and Supplementary Figure 2), which suggested that the *in vitro* EA.hy926 cells capillary formation was inhibited. F-actin structure was stained by FITC–phalloidin assay. Plumbagin (5 μM) suppressed the changes in cell morphology and actin remodeling in the Ea.hy926 cells that was induced by co-culturing them with SMMC-7721 cells (Figure 1E).

### Effects of plumbagin on the mRNA expression of the angiogenesis indicators VEGF-A/VEGFR-2, ANG2/TIE2 and FLT1 *in vitro*

**Figure 2:**
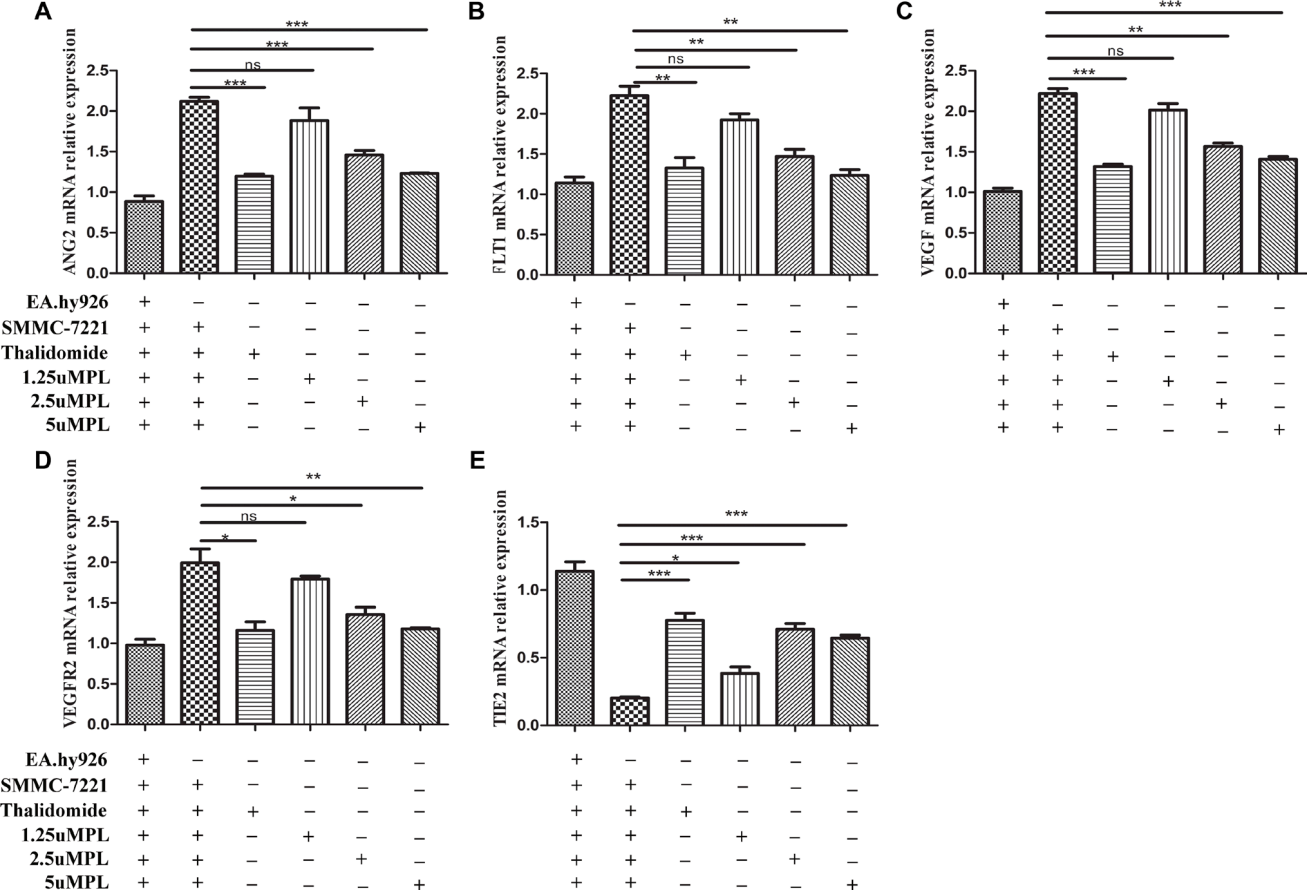
Effects of plumbagin on the mRNA expression of VEGF-A, VEGFR-2, ang2, Tie2, and FLT1 genes in SMMC-7721 cells co-cultured with EA.hy926 cells After being exposed to the indicated concentrations of plumbagin (1.25, 2.5, 5 μM), Thalidomide (25 μM) for 24 h, the mRNA expression of ang2 (**A**) FLT1 (**B**) VEGF-A (**C**) VEGFR-2 (**D**) and Tie2 (**E**) were analyzed using quantitative real-time PCR. β-actin was used as an internal control. Thalidomide (25 μM) was used as the positive control. The data represent the mean values of three experiments ± SE. *P < 0.05, **P < 0.01, ***p < 0.001 compared to co-culture with the hy926 cells.

VEGF is a main regulator of angiogenesis. studies showed that the VEGF-A/VEGFR-2/MEK1/ERK1/2 signaling pathway plays a central role in Hepatocellular carcinoma [15]. Ang-2 bind to the Tie2 tyrosine-kinase receptor, which was reported to promote angiogenesis and tumor growth [16]. Thus, combined inhibition of VEGF and Ang-2 may have complementary effects on inhibiting HCC angiogenesis. On the basis that plumbagin treatment was able to significantly inhibit the angiogenesis induced in SMMC-7721 cells that were co-cultured with EA.hy926 cells, the present study indicates that plumbagin dose-dependently inhibits VEGF-A, VEGFR-2, ang2, and FLT1. (Figure 2A–2E).

### ELISA way detect the bFGF, CTGF, ET-1, VEGF in the plumbagin-treated cell co-culture supernatants

**Figure 3:**
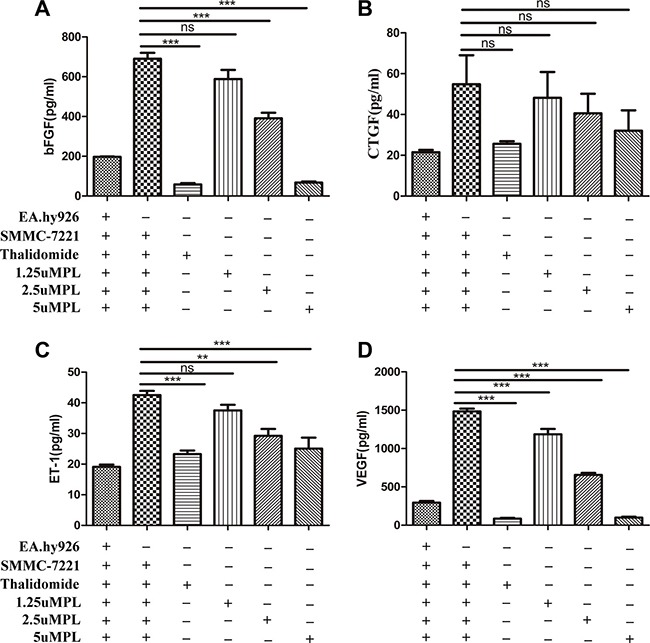
Plumbagin dose-dependently inhibits bFGF, ET-1, and VEGF *in vitro* (**A**–**D**) After incubation with plumbagin, the co-cultured cells (SMMC-7721 cells and EA.hy926 cells) were stimulated with various concentrations of plumbagin (1.25, 2.5, 5 μM), Thalidomide (25 μM) for 24 h. Thalidomide (25 μM) was used as the positive control, and the bFGF, CTGF, ET-1, and VEGF cytokines in the culture supernatants were measured using ELISA. The statistical analysis showed that plumbagin suppressed the bFGF, ET-1, and VEGF production in a dose-dependent manner. The data represent the mean values of three experiments ± SE. *P < 0.05, **P < 0.01, ***p < 0.001 compared to co-culture with hy926 cells.

The result shown that treatment with plumbagin observably suppressed the secretion of bFGF, CTGF, ET-1, VEGF from SMMC-7721 cells co-cultured with EA.hy926 cells into the culture supernatant. Specifically, *in vitro* treatment with plumbagin (1.25, 2.5, 5 μM) dose-dependently inhibited bFGF (588.13 ± 72.12, 391.00 ± 43.93, 337.04 ± 42.27), ET-1 (37.50 ± 2.88, 29.23 ± 3.51, 25.05 ± 5.57), VEGF (1186.50 ± 109.73, 656.22 ± 45.41, 499.70 ± 80.07), respectively (Figure 3A–3D). The results revealed that endothelial cells may play a important role as a target for angiogenesis inhibition by plumbagin.

### Plumbagin inhibits the activation of the PI3K-Akt, VEGF/KDR, Angiopoietin/Tie2 signaling pathways and VEGFR1/R2 in SMMC-7721 cells co-cultured with EA.hy926 cells

**Figure 4:**
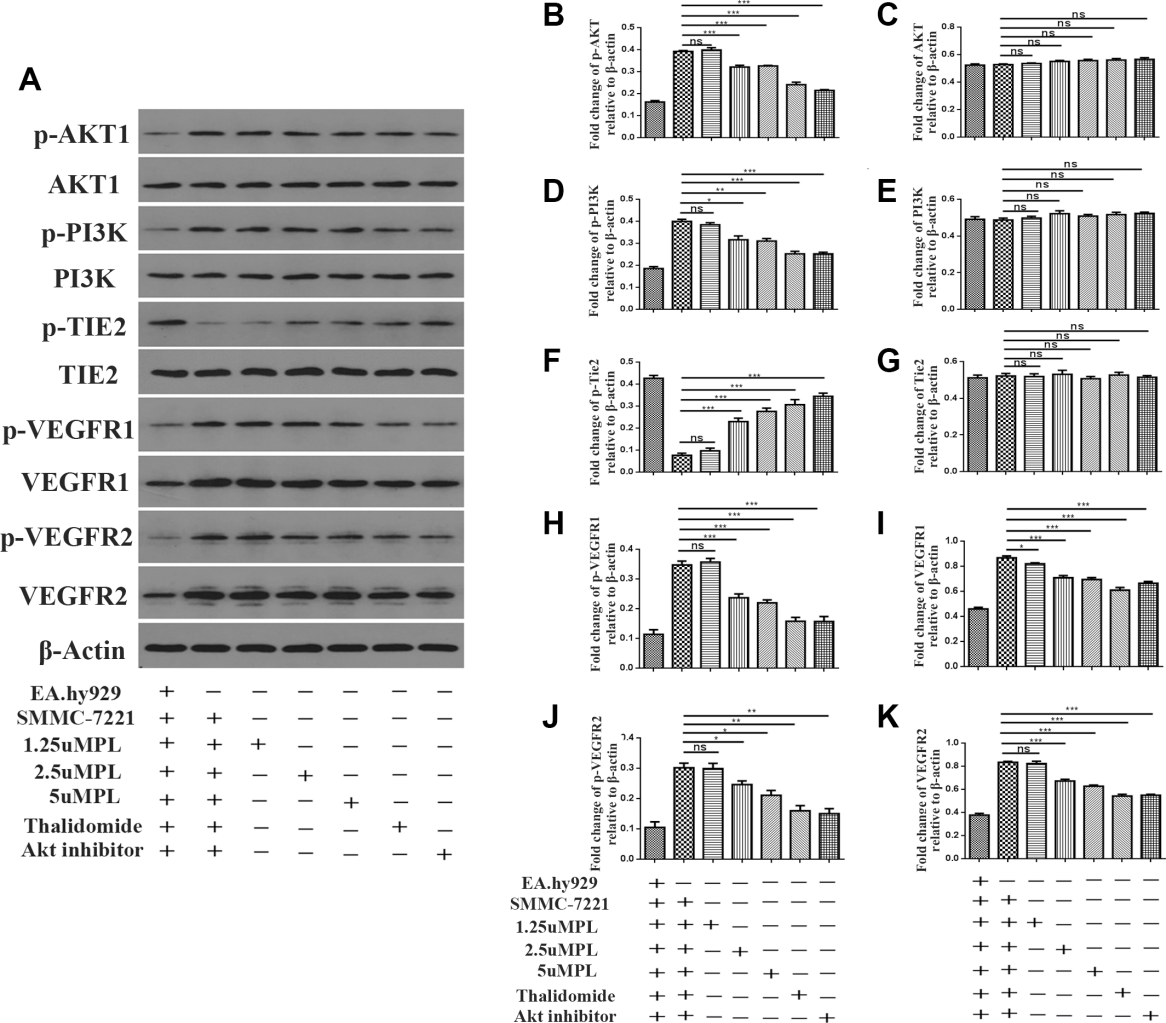
Plumbagin inhibits Angiopoietins through inactivation of p-AKT, AKT1, p-PI3K, PI3K, VEGFR1, VEGFR2, p-vegfr1, p-VEGFR2 and activation of p-TIE2 and TIE2 After co-culture, the hy926 cells were exposed to different concentrations of plumbagin for 48 h. (**A**) Plumbagin inhibits protein expression. (**B**–**K)** The expression of p-AKT, AKT1, p-PI3K, PI3K, p-TIE2, TIE2, VEGFR1, VEGFR2, p-vegfr1, p-VEGFR2 proteins in the cells was analyzed by western blotting using specific antibodies. Thalidomide (25 μM) was used as the positive control, GSK690693 (10 μM) was used as the Akt Inhibitor group. The data represent the mean values of three experiments ± SE.*P < 0.05, **P < 0.01, ***p < 0.001 compared to co-culture with hy926 cells.

To illuminate whether plumbagin was able to inhibiting the angiogenesis induced by co-culture of EA.hy926 cells with SMMC-7721 cells by blocking of the PI3K-Akt, VEGF/KDR,Angiopoietin/Tie2 signaling pathways and VEGFR1/R2 in the EA.hy926 cells, the levels of these proteins in cells exposed to diverse concentrations of plumbagin were detected using western blotting with antibodies specific for the targeted proteins. As shown in (Figure 4A–4K and Supplementary Figure 3), when the SMMC-7721 cells were co-cultured with the EA.hy926 cells, the levels of phosphorylation/activation of PI3K-Akt, KDR increased. Furthermore, the expression of the above proteins in the EA.hy926 cells was significantly downregulated in a dose-dependent manner by the plumbagin treatment. These results revealed that the overexpression of PI3K-Akt, VEGF/KDR, Angiopoietins /Tie2 and VEGFR1/R2 may be significantly suppressed by plumbagin treatment *in vitro*.

### Plumbain inhibit SMMC-7221 cells growth of xenografted tumour *in vivo*

**Figure 5:**
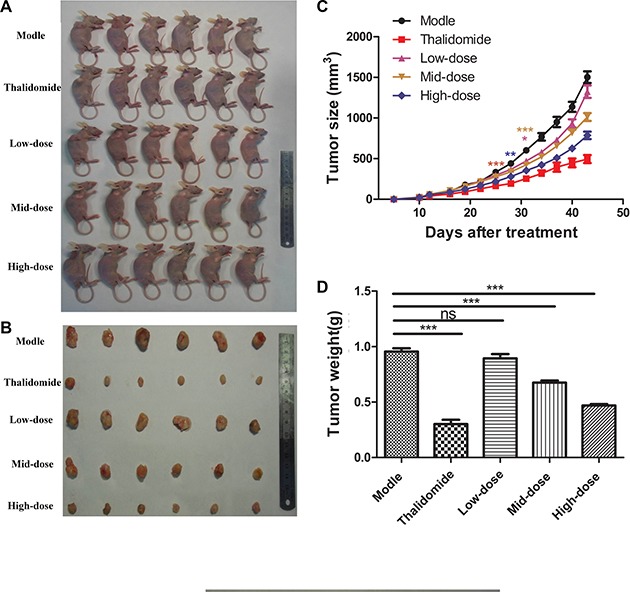
Plumbagin inhibits tumor growth *in vivo* (**A**) SMMC-7721 cells were subcutaneously injected into the right backs of female nude mice. After the tumors had grown to approximately 150 mm^3^–200 mm^3^, the mice received plumbagin (1.25 mg/kg/d, 2.5 mg/kg/d, or 5 mg/kg/d), saline (0.5 ml/d) or Thalidomide (200 mg/kg/d) every day for 30 d. (**B**) Photographs were taken of representative mice 30 days after treatment. (**C**) The tumor volumes are presented as the means ± SD of six mice as described in (A). (**D**) The tumor weights were measured 30 days after treatment. The data represent the mean values of six mice ± SE.*P < 0.05, **P < 0.01, ***p < 0.001 compared to the model group.

To evaluate the plumbagin antitumour *in vivo*,we used a SMMC-7221 tumour xenograft mouse model. Photographs confirmed that tumour formation in the nude mice, and tumours sizes were measured using calipers every three days until the end of experiment to monitor the *in vivo* therapeutic efficacy. As shown in Figure 5A–5B.

Growth curves of xenografted tumours showed that tumours of treatment with plumbagin observably inhibited the primary tumor growth compared with control group, especially at the 5 mg/kg/d dose (*P* < 0.05). (Figure 5C–5D). A slight time-dependent increase in the RTV was observed in the groups treated with saline (0.5 ml/d), plumbagin (1.25 mg/kg/d, 2.5 mg/kg/d, 5 mg/kg/d), and Thalidomide (200 mg/kg/d), resulting in average RTV values of 27.42, 21.64, 17.26, 15.03 and 13.46, respectively, on day 43. The results in Table 1 also shown that treatment with plumbagin observably inhibited the primary tumor growth compared with control group, especially at the 5 mg/kg/d dose (*P* < 0.05). The relative TIR obtained with plumbagin was 6.40% for 1.25 mg/kg/d, 29.29% for 2.5 mg/kg/d, and 50.94% for 5 mg/kg/d, respectively (Table 1).

**Table 1:** Inhibitory effect of plumbagin on the growth of human liver cancer SMMC-7221 cell xenografts in nude mice

Group	Dose(mg/kg/day)	Body weight (g)		Increase in body weight (g)	TV (mm^3^)		RTV	TIR(%)
		Before	After		Before	After		
Model	saline	17.88	18.58	0.95	52.85	1502.68	27.42	0.00
Thalidomide	200	17.98	19.68	0.30	40.06	494.91	13.46	68.36*
low-dose	1.25	18.00	19.78	0.89	59.64	1324.43	21.64	6.40
mid-dose	2.5	18.03	19.78	0.67	61.01	1013.20	17.26	29.29
high-dose	5	17.75	19.13	0.46	55.12	786.42	15.03	50.94*

The data are presented as the means ± SD (*n* = 6).

*significantly different from the model group, *p* < 0.05.

### Effect of plumbagin on tumor angiogenesis *in vivo*

**Figure 6:**
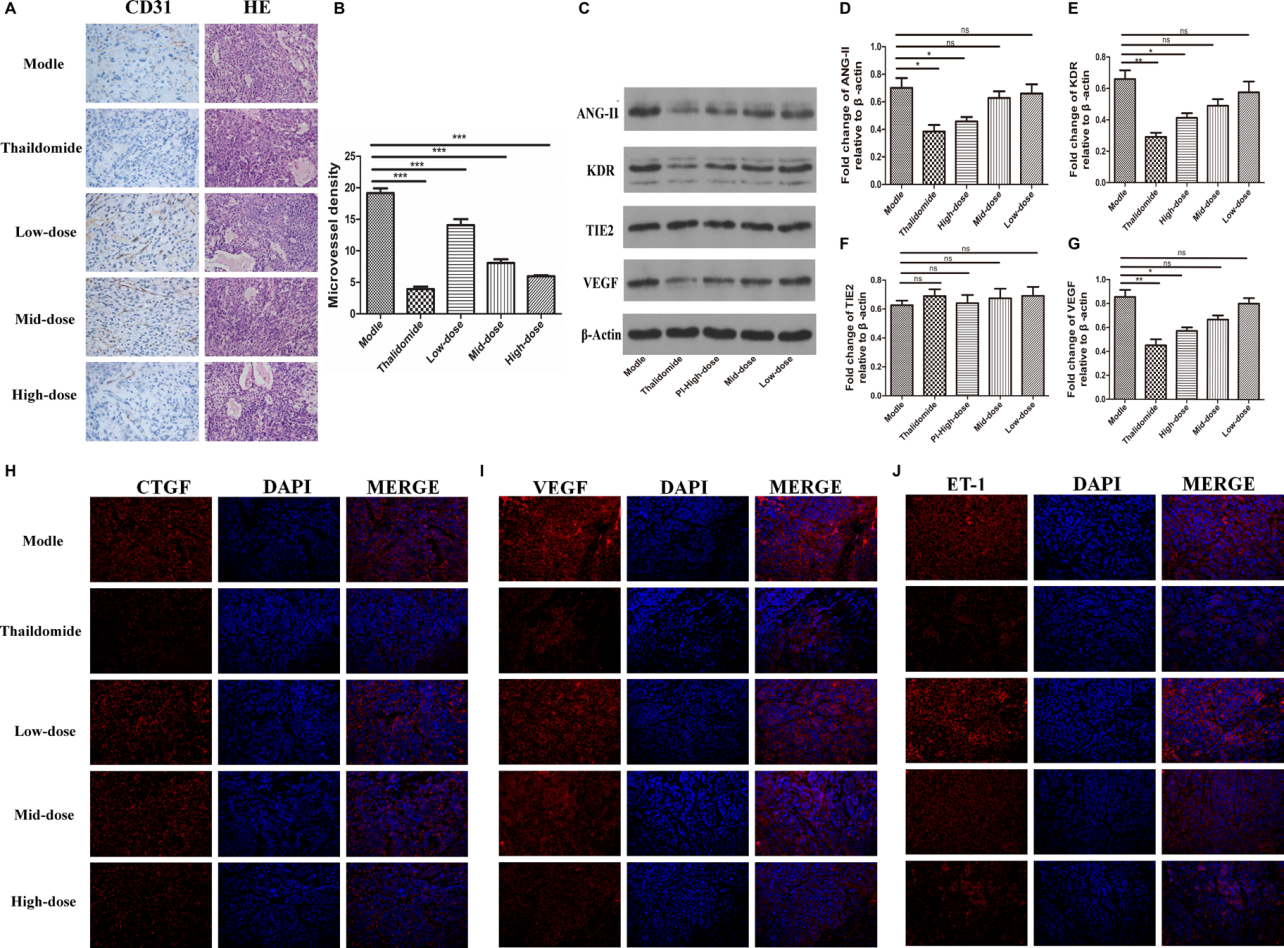
Plumbagin suppresses VEGF expression and tumor angiogenesis *in vivo* (**A)** Plumbagin inhibits tumor angiogenesis *in vivo*. The histological analysis of the livers of mice treated with plumbagin for 30 days after treatment is shown (original magnification ×200). Tumor angiogenesis was assessed by IHC using an antibody to CD31 on sections of tumors from mice treated with plumbagin (1.25 mg/kg/d, 2.5 mg/kg/d, 5 mg/kg/d), saline (0.5 ml/d) and thalidomide (200 mg/kg/d). (**B**) The MVD was the average of the vessel counts obtained in the five sections from each group. (**C**–**G**) The expression of the VEGF, ANG-II, Tie2, and KDR proteins was analyzed in the SMMC-7221 cells by western blotting using specific antibodies. An anti-b-actin antibody was used to confirm equivalent protein loading. (**H**–**J**) Immunofluorescence analyses of the expression of ET-1, VEGF, and CTGF in the murine liver orthotropic model and experimental liver-metastasis model using smmc-7221 cells were conducted to determine the effect of plumbagin *in vivo*. The data represent the mean values of six mice ± SE. *P < 0.05, **P < 0.01, ***p < 0.001 compared to the model group.

Haematoxylin and eosin staining showed that the damaged cells. (Figure 6A, right panel lane). Compared with the model group, there were spaces between the cells and pyknotic nuclei in the three doses of plumbagin and thalidomide treatment groups. As a result, the plumbagin and thalidomide groups were found to ameliorate the severity of the tumors. In addition, the high-dose plumbagin treatment was the most efficacious. We further investigated the effect of plumbagin on tumor angiogenesis *in vivo* and determined the expression of CD31. The IHC analysis showed that the positive staining of CD31 was markedly lower in the tumors treated with plumbagin than in the model group (Figure 6A, left panel lane). The tumor-associated neovascularization as indicated by MVD was quantified. The MVD was markedly lower in the tumors treated with plumbagin than in the model group (5.96 vs 19.15, Figure 6B). These results indicate that plumbagin inhibits tumor angiogenesis *in vivo*.

### Effect of plumbagin on tumor angiogenesis by VEGF/KDR, ANG2/TIE2, ET-1 and CTGF signal pathways *in vivo*

The two signal transduction pathways, VEGF/KDR and ANG2/TIE2, play important roles in the process of angiogenesis in tumor growth and metastasis. As shown in (Figure 6C–6F) there was a marked decline in the expression of the VEGF/KDR and Ang2 proteins in the tumor tissues from both the plumbagin and thalidomide treatment groups (*P* < 0.05). There was not a difference between high-dose plumbagin and thalidomide treatment groups for the tumor VEGF/KDR and ANG2/TIE2 levels. Immunofluorescence further verified the ET-1, VEGF, and CTGF expression. The results also demonstrated that plumbagin has an remarkable therapeutic potential for human HCC.

## DISCUSSION

The goal of this study was to examine whether plumbagin could inhibit the angiogenesis mediated growth of HCC carcinoma cells through abrogation of the PI3K/AKT pathway in an orthotopic mouse model. Our results suggest that plumbagin is a potent angiogenesis inhibitor and inhibits multiple steps of angiogenesis, including endothelial cell viability, migration, invasion, differentiation into capillary like structures and angiogenic factors. Plumbagin was found to exert its anti-angiogenic effects by targeting the PI3K/AKT signaling cascade in endothelial HCC cells.

Endothelial cells, which are the major components of blood vessels, unnormal condition the angiogensis more rapidly. VEGF is perhaps the most extensively studied angiogenic cytokine and has successfully been developed as a therapeutic target for the inhibition of angiogenesis in HCC. Previous study have found tumor cell lines express VEGF and its receptorsVEGFR1/2 have been observed to be expressed in endothelial cells [17]. VEGF/KDR and ANG/Tie2 two signal pathway play an important role in the process of angiogenesis in HCC growth and metastasis [18]. Especially, the environment of hypoxia in HCC stimulate tumor cell to secret VEGF,which activates its receptor KDR and result in endothelial cells proliferation, migration and tubal formation [19, 20]. At the same time, angiogenesis involve dissolution of the vascular basal membrane, increased vascular permeability and the degradation of extracellular matrix. We analyzed the rate of proliferation and the viability of EA.hy926 cells when they were co-cultured with SMMC-7721 or Hep3B cells. Plumbagin had an ability to reduce the angiogenic cytokine-induced proliferation of the endothelial cells to abrogate the formation of tumor vasculature. Treatment of the EA.hy926 cells with plumbagin effectively abrogated the angiogenic cytokine-induced migration, invasion, and capillary-like structures formation *in vitro*.

Angiogenic cytokines such as basic fibroblast growth factor (bFGF), connective tissue growth factor (CTGF), endothelia (ET-1) and angiopoietins (ANGs) regulate tumor angiogenesis. Under physiological conditions, Ang-2 is only weakly expressed in endothelial cells (ECs). During tumor growth the Ang2 overexpressed in endothelial cells [21]. ANG2 binds to TIE2 and acts as a negative regulator of ANG1/Tie2 signaling during angiogenesis, thereby controlling the responsiveness of the endothelial cells to exogenous cytokines [22]. We observed for the first time that plumbagin can significantly suppress the production of angiogenic cytokines (VEGF, bFGF, CTGF, and ET-1) and regulate the ANG1/2 balance when the SMMC-7721 were co-cultured with EA.hy926 cells or in the tumors in an orthotropic HCC mouse model.

**Figure 7:**
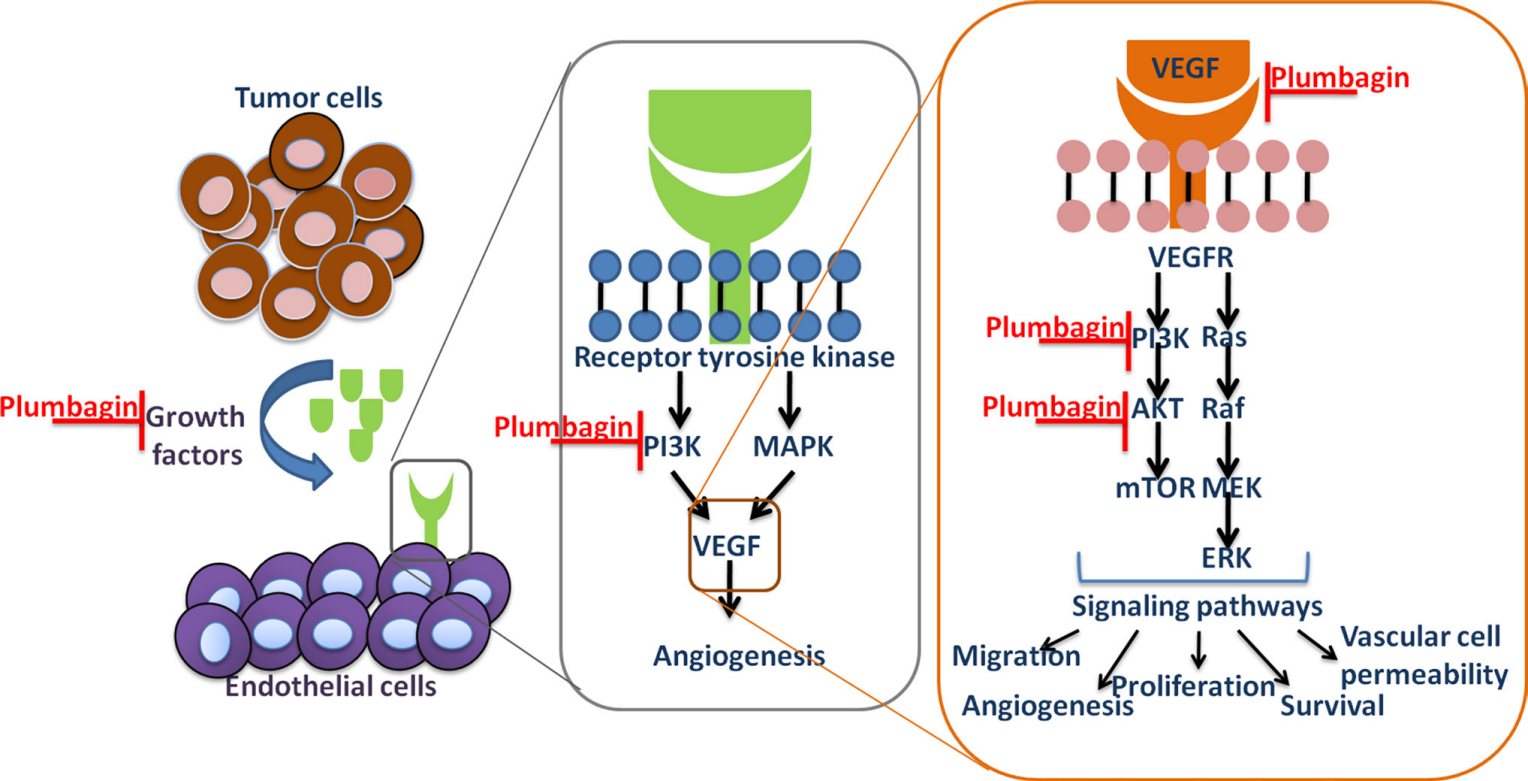
Tumor cells secrete pro-angiogenic growth factors that bind to receptors on dormant endothelial cells (ECs), which lead to vasodilation and an increase in vessel permeability The ECs migrate and proliferate to form new branches from the pre-existing vasculature by detaching from the extracellular matrix and basement membrane.

The PI3K/AKT pathway is involved in the regulation of multiple cellular processes, including cell proliferation, migration, invasion and survival. In many cancers this pathway is overactive, reducing apoptosis, allowing proliferation and thus enhanced signaling through this pathway is a significant contributor to new blood vessel formation [23, 24]. GSK690693 was used as the Akt inhibitor is effective in the treatment of cancer. We tested the *in vivo* effects of GSK690693 in SMMC-7721 or Hep3B cells when they were co-cultured with EA.hy926 cells that develop angiogenesis of HCC .We found that treatment with GSK690693 substantially reduced the activation of PI3K/AKT in endothelial cells in a time-dependent manner. We further noticed that plumbagin treatment also inhibit the expression of phospho-PI3K and AKT Figure 7, We also demonstrate for the first time the potential of plumbagin to inhibit tumor-induced angiogenesis in HCC patient xenografts implanted in BALB/c mice. To the best of our knowledge, no prior studies with plumbagin have been conducted in HCC mouse models, and our observations clearly indicate that plumbagin treatment of HCC through the abrogation of PI3K/AKT pathway.

## MATERIALS AND METHODS

### Cell culture

The human hepatoma cell line SMMC-7721, the human endothelial cell line EA.hy926, the human hepatoma cell line Hep3B cell (Shanghai Institutes for Biological Sciences, Shanghai, China), and the co-cultured (EA.hy926 cells , SMMC-7721 and Hep3B cells: various experimental approaches used a different co-culture method) cells were maintained in DMEM medium (HyClone, Utah, USA) containing 10% fetal bovine serum (FBS) (Invitrogen, CA, USA), 100 U/ml penicillin and 100 μg/ml streptomycin. For experiments under hypoxic conditions, the cells were cultured in a hypoxia chamber adjusted to 5% CO_2_ and 1% O_2_ at 37°C.

### Cell viability assay

SMMC-7721 cells and EA.hy926 cells were plated at a density of 5 × 10^3^ cell/well in 96-well plates. After 24 h of culture, the SMMC-7721 cells were exposed to various concentrations of thalidomide (25, 50, 75, 100 or 200 μg/ml) for 24 h in a 5% CO_2_ incubator at 37°C. At the same time, EA.hy926 cells and SMMC-7721 cells were exposed to various concentrations of plumbagin (1.25, 2.5 or 5 μM) for 12, 24 or 48 h in a 5% CO_2_ incubator at 37°C. Then, MTT (1 mg/mL) (Sigma, St. Louis, Mo) was added to the cells, and they were incubated for an additional 4 hours at 37°C. The supernatant fluid was removed, and 100 μL of dimethylsulfoxide (DMSO) (Sigma, St. Louis, Mo) was added to each well. The absorbance (OD value) was measured using a Multiskan MK3 micro enzyme-linked immunosorbent assay reader (Thermo Scientific, MA, USA) at a wavelength of 570 nanometers (nm).

### Migration and invasion

The EA.hy926 cells were collected in medium containing 10% fetal bovine serum at a final concentration of 2 × 10^5^ cell/mL and pre-incubated for 24 h in 24-well plates. Then, SMMC-7721 cells or Hep3B cells were plated in the upper wells of transwell BD BioCoat^™^ Matrigel^™^invasion chambers (pore size: 8 μm, BD Biosciences). Serum-free medium containing various concentrations of plumbagin (1.25, 2.5 or 5 μM) was added, and the cells were pretreated for 48 h in 24-well plates. In a second series of experiments, the SMMC-7721 cells or Hep3B cells were collected in medium containing 10% fetal bovine serum at a final concentration of 2 × 10^5^ cell/mL and pre-incubated for 24 h in 24-well plates. Then, hy926 cells were plated in the upper well of the transwell migration inserts (pore size: 8 μm, BD Biosciences). Serum-free medium containing various concentrations of plumbagin (1.25, 2.5, 5 μM) was added, and the cells were pretreated for 48 h in 24-well plates. Thalidomide (25 μM) was used as the positive control.

GSK690693 (10 μM) was used as the Akt inhibitor group. Following incubation for 24 h, the invading cells that had migrated to the lower surface were stained with hematoxylin and eosin, and then quantified by manual counting. Five randomly chosen fields were analyzed for each group.

### Real-Time PCR analysis

For the co-culture with the hy926 cells, the SMMC-7721 cells were collected in medium containing 10% fetal bovine serum at a final concentration of 2 × 10^5^ cell/mL and incubated for 24 h in 24-well plates. Then, hy926 cells in serum-free medium were added to the upper well of the transwells at a final concentration of 2 × 10^4^ cell/mL. Various concentrations of plumbagin (1.25, 2.5 or 5 μM), Thalidomide (25 μM) were added, and the cells were incubated for 48 h in 24-well plates. The RNA was isolated with TRIzol reagent (Takara, Japan) according to the manufacturer's instructions, and cDNA was synthesized with Prime Script RT reagent Kit (Takara, Japan) according to the manufacturer's protocol and used as the template for quantitative PCR. Real-time PCR was performed using SYBR Premix Ex TaqII(TaKaRa, Japan) on a Chromo4 continuous fluorescence detector with Multiskan MK3 (Thermo Scientific, MA, USA). Thalidomide (25 μM) was used as the positive control. The mRNA levels of VEGF-A, VEGFR-2, ang2, Tie2, FLT1 were evaluated. The primer sets used for the PCR amplifications were as follows:

VEGF-A (forward: 5′-AAGGAGGAGGGCAGAA TCAT-3′, reverse: 5′-ATCTGCATGGTGATGTTGGA-3′), VEGFR-2 (forward: 5′-AGCGATGGCCTCTTCTGTA A-3′, reverse: 5′-ACACGACTCCATGTTGGTCA-3′), ang2 (forward: 5′-CAGGAGGCTGGTGGTTTGAT-3′, reverse: 5′-AGGTGGACTGGGATGTTTAG-3′), Tie2 (forward: 5′-GCTTGGACCCTTAGTGACATTCT-3′, reverse: 5′-GCCTTGAACCTTGTAACGGATAG-3′), FLT1 (forward: 5′-GTGTAAGGAGTGGACCATCATT C-3′, reverse: 5′-TTCTCAGTCGCAGGTAACCCATC-3′, and β-actin (forward: 5′-AGCGAGCATCCCCCAAAG TT-3′, reverse: 5′-GGGCACGAAGGCTCATCATT-3′).

### ELISA

For the co-cultures, SMMC-7721 cells at a final concentration of 2 × 10^5^ cell/mL were plated in 24-well plates in medium containing 10% fetal bovine serum and incubated for 24 h. Then, hy926 cells at a final concentration of 2 × 10^4^ cell/mL in serum-free medium were plated into the upper compartment of the transwells, and various concentrations of plumbagin(1.25, 2.5 or 5 μM) ,Thalidomide (25 μM) were added for 48 h. The supernatants were collected. The production of the cytokines bFGF, CTGF, ET-1, and VEGF was analyzed using ELISA kits according to the manufacturer's protocol. The absorbance (OD value) was measured using Multiskan MK3 micro enzyme-linked immunosorbent assay reader (Thermo Scientific, MA, USA) at a wavelength of 570 nanometers (nm).

### Western blotting

For the co-cultures, SMMC-7721 cells at a final concentration of 2 × 10^5^ cell/mL were plated in 24-well plates in medium containing 10% fetal bovine serum and incubated for 24 h. Then, hy926 cells at a final concentration of 2 × 10^4^ cell/mL in serum-free medium were plated into the upper compartments of the transwells, and various concentrations of plumbagin(1.25, 2.5 or 5 μM),Thalidomide (25 μM) and GSK690693(10 μM)were added for 48 h. The hy926 cells were then lysed in RIPA buffer containing PMSF. The protein samples (20 μg) were electrophoresed on 6%–12% denaturing sodium dodecylsulfate (SDS) gels and transferred to PVDF membranes (Millipore, MA, USA). The blots were incubated with specific primary antibodies as follows: the p-PI3K polyclonal antibody, p-tie2 rabbit polyclonal antibody, and KDR mouse monoclonal antibody were purchased from Santa Cruz (Santa Cruz, CA). The AKT, p-AKT, VEGFR2and PI3K rabbit antibodies were from Cell Signaling Technology Corp (Beverly, MA, USA). VEGFR1 rabbit antibodies were from Abcam (Abcam, USA), p-vegfr1, p-VEGFR2 rabbit antibodies were from RD (MA, and USA).The β-actin and tie2 mouse monoclonal antibodies were from Boste (Boste, Wuhan, CN). The primary antibody signals were detected using horseradish peroxidase-conjugated secondary antibodies (Boste, Wuhan, and CN). The anti-β-actin antibody was used as an internal loading control. The antigen-antibody complexes were visualized using the ECL system (Amersham Biosciences, Piscataway, NJ).

### Immunofluorescence

SMMC-7721 cells were cultured on coverslips. FITC–phalloidin was used to probe the samples for 1 h to analyze the actin remodeling. Additional coverslips were incubated overnight at 4°C with primary antibodies (diluted 1:50) directed against E-cadherin and vimentin. After the cells were washed, they were exposed to FITC-conjugated secondary antibodies (1:1000; Invitrogen, Carlsbad, CA, M30101, L42001), and then stained with DAPI for 20 min. The coverslips were washed and mounted. All tissue samples were embedded in paraffin blocks and then sectioned. Immunofluorescence staining was performed on 6 μm paraffin sections of the tumor tissue, which was blocked with 20% goat serum and then incubated with anti-mouse vegf mAb 1/1000 (Abcam, USA), anti-mouse ctgf 1/1000 (Abcam, USA) or anti-mouse ET-1 1/1000 (Abcam, USA) at 4°C overnight. To detect the bound antibodies, CY3-labeled goat anti-rabbit IgG at 1/1000 (Abcam, USA) or FITC-labeled goat anti-mouse at 1/200 (Abcam, USA) was used at 22°C for 45 min. Nuclear staining was performed with DAPI at a 1/1000 dilution (Abcam, USA) at 22°C for 10 min. At least three sections per mouse were examined for each immunostaining condition. Images were acquired using a confocal microscope.

### Angiogenesis assay

SMMC-7721 cells were collected in medium containing 10% fetal bovine serum at a final concentration of 2 × 10^4^ cell/mL and pretreated for 24 h in 24-well plates. Matrigel was melted at 4°C overnight then added to cold 24-well plates and incubated for an additional 40 min at 37°C. Subsequently, the supernatant was collected. Logarithmic phase EA.hy926 cells at 6–8 × 10^5^ to which various concentrations of plumbagin (1.25, 2.5, 5 μM) Thalidomide (25 μM) and GSK690693 (10 μM) had been added were incubated for 6 h at 37°C. The capillary structures were examined using an Olympus microscope, and statistical analysis was performed using Image-Pro Plus software.

### Plumbagin therapy in a mouse xenograft model of HCC

Four- to six-week-old female BALB/c nude mice (Specific Pathogen Free, SPF) were bred in the Animal Experimental Center of the Shanghai Institutes for Biological Sciences (Shanghai, China). SMMC-7721 cells (2.5 × 10^7^ SMMC-7721) were injected subcutaneously (s.c.) into the right flanks of the mice. Two weeks later, when the size of tumors had reached approximately 150–200 mm^3^, the mice were randomly divided into 5 groups (six mice/group) and treated daily for 30 d by intraperitoneal injection with plumbagin (1.25 mg/kg/d, 2.5 mg/kg/d, or 5 mg/kg/d), saline (0.5 ml/d) or Thalidomide (200 mg/kg/d). The tumor volumes (V) were calculated according to the following formula: V (mm^3^) =1/2 ab^2^ (a: relatively shorter diameter, b: relatively longer diameter). The animals were killed after 30 days of treatment, and their tumors were weighed and harvested for histological analysis, immunohistochemistry (IHC) and western blot analysis. The tumor inhibitory rate (IR) was calculated as IR = (C−T)/C × 100%, where T is the average tumor weight of the experimental group, and C is the average tumor weight of the model group. The evaluation criteria were: IR < 30% (invalid); IR ≥ 30% and *P* < 0.05 (valid).

### HE and immunohistochemistry

The tumor tissues were fixed in 10% formalin. The tumors were embedded in paraffin and stained with hematoxylin and eosin (H&E). The tissue sections were deparaffinized, rehydrated and boiled in 0.01 M sodium citrate for antigen retrieval. The endogenous peroxidase activity was quenched. The sections were incubated with anti-CD31 (1:50, abcam, Cambridge, MA) overnight at 4°C. The tumor sections were incubated with biotinylated secondary antibodies and a streptavidin-biotin complex (Zsbio, Wuhan, China). The staining was visualized using DAB (Boster,Wuhan, China). Representative photos were taken using an Olympus camera (Nikon Instruments, Melvil).

## CONCLUSIONS

Thus, overall, our experimental findings clearly indicated that the anti-cancer effects of plumbagin in HCC are mediated through the mitigation of PI3K/AKT signaling cascade and thus provide a strong rationale for pursuing the use of plumbagin in the treatment of HCC and other malignancies where angiogenesis is the key contributor to disease progression. The present study provided a basis for novel therapeutic options for the treatment of HCC patients.

## SUPPLEMENTARY MATERIALS FIGURES



## References

[1] Weis SM, Cheresh DA (2011). Tumor angiogenesis: molecular pathways and therapeutic targets. Nat Med.

[2] Amini A, S Masoumi Moghaddam, Morris DL, Pourgholami MH (2012). The critical role of vascular endothelial growth factor in tumor angiogenesis. Curr Cancer Drug Targets.

[3] Shojaei F (2012). Anti-angiogenesis therapy in cancer: current challenges and future perspectives. Cancer Lett.

[4] Zhu H-F, Wan D, Luo Y, Zhou J-L, Chen L, Xu X-Y (2010). Catalpol Increases Brain Angiogenesis and Up-Regulates VEGF and EPO in the Rat after Permanent Middle Cerebral Artery Occlusion. International Journal of Biological Sciences.

[5] Ng IOL, Poon RTP, Lee JMF, Fan ST, Ng M, Tso WK (2001). Microvessel density, vascular endothelial growth factor and its receptors Flt-1 and Flk-1/KDR in hepatocellular carcinoma. American Journal of Clinical Pathology.

[6] Potente M, Gerhardt H, Carmeliet P (2011). Basic and Therapeutic Aspects of Angiogenesis. Cell.

[7] Kang Z, Jiang W, Luan H, Zhao F, Zhang S (2013). Cornin induces angiogenesis through PI3K-Akt-eNOS-VEGF signaling pathway. Food and Chemical Toxicology.

[8] Padhye S, Dandawate P, Yusufi M, Ahmad A, Sarkar FH (2012). Perspectives on medicinal properties of plumbagin and its analogs. Med Res Rev.

[9] Shih YW, Lee YC, Wu PF, Lee YB, Chiang TA (2009). Plumbagin inhibits invasion and migration of liver cancer HepG2 cells by decreasing productions of matrix metalloproteinase-2 and urokinase-plasminogen activator. Hepatol Res.

[10] Hwang GH, Ryu JM, Jeon YJ, Choi J, Han HJ, Lee Y-M, Lee S, Bae J-S, Jung J-W, Chang W, Kim LK, Jee J-G, Lee MY (2015). The role of thioredoxin reductase and glutathione reductase in plumbagin-induced, reactive oxygen species-mediated apoptosis in cancer cell lines. European Journal of Pharmacology.

[11] Kuo YH, Chang CI, Li SY, Chou CJ, Chen CF, Kuo YH, Lee KH (1997). Cytotoxic constituents from the stems of Diospyros maritima. Planta Med.

[12] Parimala R, Sachdanandam P (1993). Effect of Plumbagin on some glucose metabolising enzymes studied in rats in experimental hepatoma. Mol Cell Biochem.

[13] Sinha S, Pal K, Elkhanany A, Dutta S, Cao Y, Mondal G, Iyer S, Somasundaram V, Couch FJ, Shridhar V, Bhattacharya R, Mukhopadhyay D, Srinivas P (2013). Plumbagin inhibits tumorigenesis and angiogenesis of ovarian cancer cells in vivo. International Journal of Cancer.

[14] Ferrara N (2002). VEGF and the quest for tumour angiogenesis factors. Nature Reviews Cancer.

[15] Bento LW, Zhang Z, Imai A, Nor F, Dong Z, Shi S, Araujo FB, Nor JE (2013). Endothelial Differentiation of SHED Requires MEK1/ERK Signaling. Journal of Dental Research.

[16] Llovet JM, Pena CEA, Lathia CD, Shan M, Meinhardt G, Bruix J, Grp SIS (2012). Plasma Biomarkers as Predictors of Outcome in Patients with Advanced Hepatocellular Carcinoma. Clinical Cancer Research.

[17] Ferrara N, Gerber HP, LeCouter J (2003). The biology of VEGF and its receptors. Nature Medicine.

[18] Beeghly-Fadiel A, Shu XO, Lu W, Long J, Cai Q, Xiang YB, Zheng Y, Zhao Z, Gu K, Gao YT, Zheng W (2011). Genetic variation in VEGF family genes and breast cancer risk: a report from the Shanghai Breast Cancer Genetics Study. Cancer Epidemiol Biomarkers Prev.

[19] Grunewald FS, Prota AE, Giese A, Ballmer-Hofer K (2010). Structure-function analysis of VEGF receptor activation and the role of coreceptors in angiogenic signaling. Biochimica Et Biophysica Acta-Proteins and Proteomics.

[20] Kieran MW, Kalluri R, Cho YJ (2012). The VEGF Pathway in Cancer and Disease: Responses, Resistance, and the Path Forward. Cold Spring Harbor Perspectives in Medicine.

[21] Vajkoczy P, Farhadi M, Gaumann A, Heidenreich R, Erber R, Wunder A, Tonn JC, Menger MD, Breier G (2002). Microtumor growth initiates angiogenic sprouting with simultaneous expression of VEGF, VEGF receptor-2, and angiopoietin-2. J Clin Invest.

[22] Augustin HG, Koh GY, Thurston G, Alitalo K (2009). Control of vascular morphogenesis and homeostasis through the angiopoietin-Tie system. Nature Reviews Molecular Cell Biology.

[23] Radisavljevic Z (2013). AKT as locus of cancer angiogenic robustness and fragility. Journal of Cellular Physiology.

[24] Ciuffreda L, Di Sanza C, Incani UC, Milella M (2010). The mTOR Pathway: A New Target in Cancer Therapy. Current Cancer Drug Targets.

