# Supplementary material for: Plumbagin restrains hepatocellular carcinoma angiogenesis by suppressing the migration and invasion of tumor-derived vascular endothelial cells
Source: Oncotarget. 2017 Jan 20;8(9):15230–41. doi: 10.18632/oncotarget.14774 (PMC5362482; doi:10.18632/oncotarget.14774)
Supplement: Supplementary file 1 [file oncotarget-08-15230-s001.pdf]

Plumbagin restrains hepatocellular carcinoma angiogenesis by suppressing the migration and invasion of tumor-derived vascular endothelial cells

Supplementary Materials

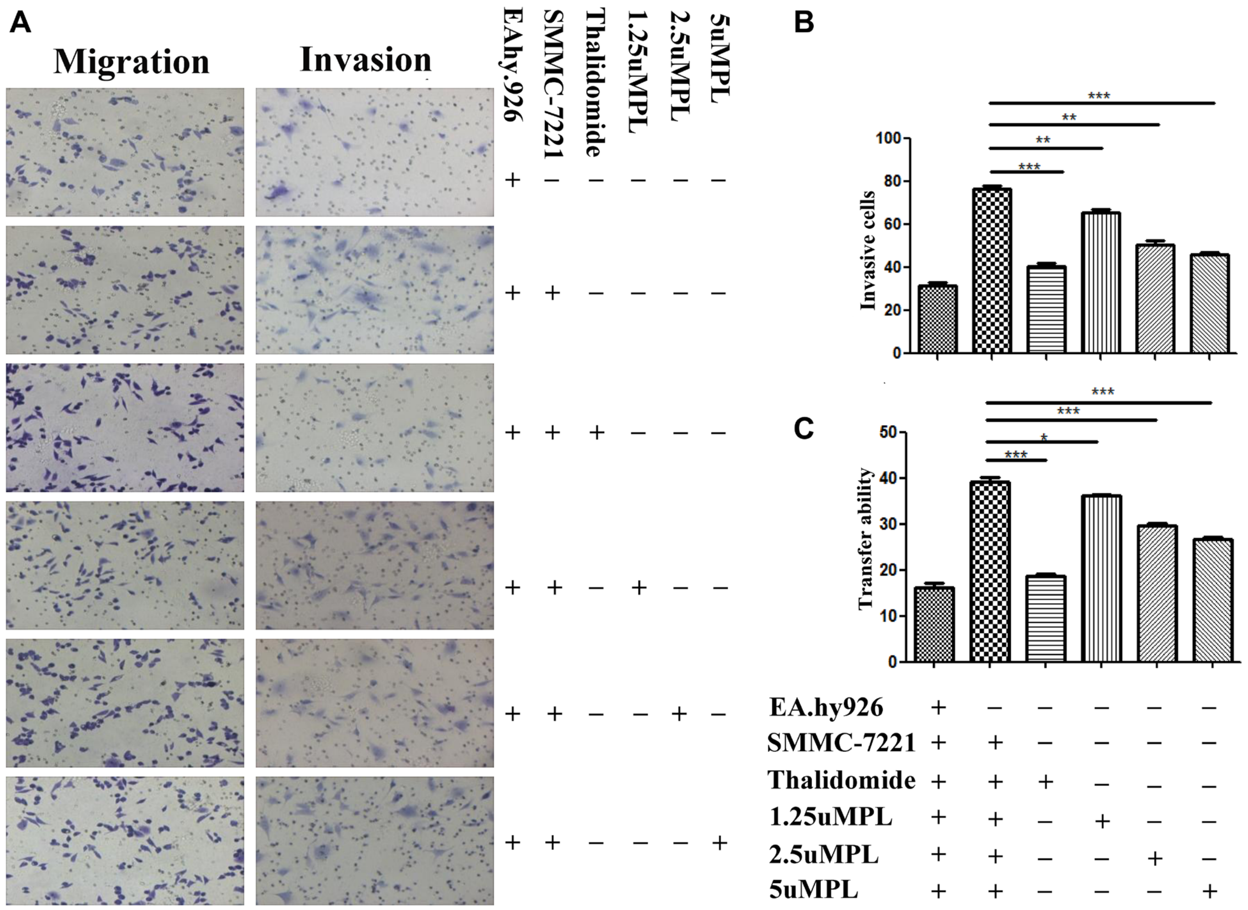

Supplementary Figure 1: Plumbagin reduced the migration and invasion of the human endothelial cell line EA.hy926 that was induced by the human hepatoma cell line smmc-7221. (**A**) Plumbagin-depleted cells (24 h) were loaded for transwell migration (left) and Matrigel invasion assays (right). (**B–C**) Migration or invasion were assessed at 24 h. Fields were counted for each well. The smmc-7221 were treated with plumbagin as indicated and migration or invasion experiments were performed as in (B–C). The data represent the mean values of three experiments ± SE. *$P < 0.05$, **$P < 0.01$, ***$p < 0.001$ compared to co-culture with hy926 cells.

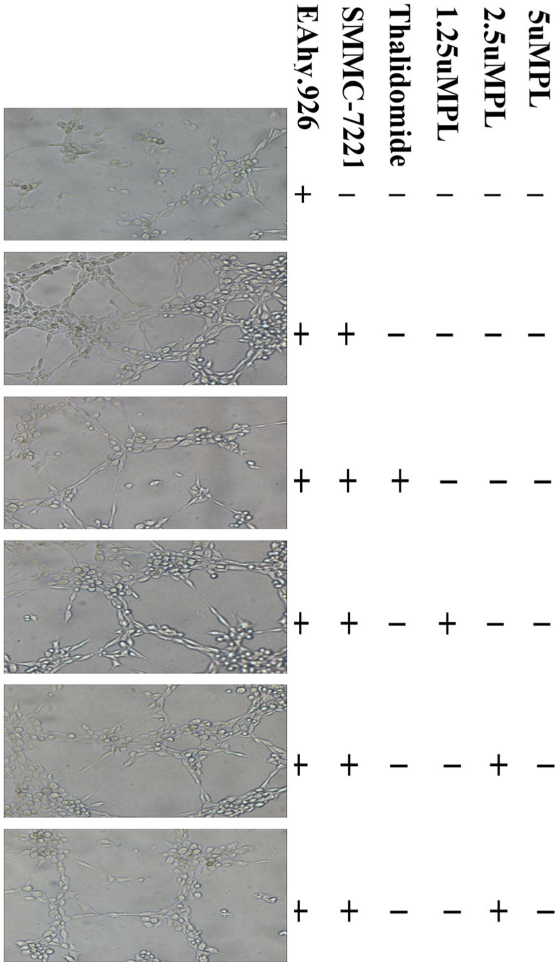

Supplementary Figure 2: The co-culture of SMMC-7721 cells and EA.hy926 cells can also spontaneously form capillary-like structures on Matrigel, and we therefore studied the effects of plumbagin on the angiogenesis in hy926 cells. Our data showed that the number and the continuity of the capillary-like structures of the hy926 cells were all dramatically inhibited by 1.25–5 μM plumbagin in a dose-dependent manner, which suggested that plumbagin inhibited the formation of tubes that was induced by the hy926 cells in vitro.Thalidomide (25μM) was used as the positive control. The data represent the mean values of three experiments ± SE.*$P < 0.05$, **$P < 0.01$, ***$p < 0.001$ compared to co-culture with hy926 cells.

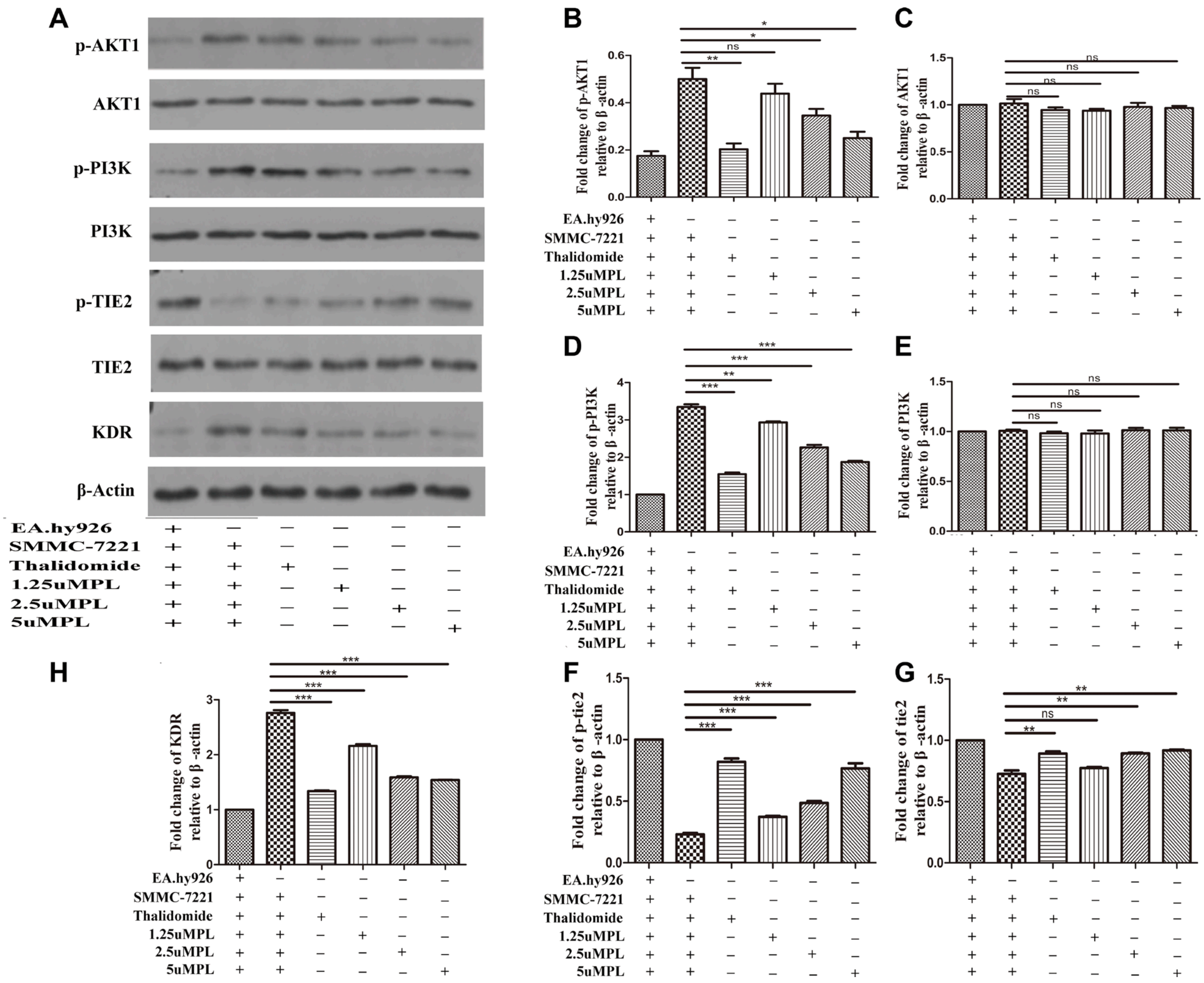

Supplementary Figure 3: Plumbagin inhibits Angiopoietins through inactivation of p-AKT, AKT1, p-PI3K, PI3K, KDR and activation of p-TIE2 and TIE2. After co-culture, the hy926 cells were exposed to different concentrations of plumbagin for 48 h. Thalidomide (25 μM) was used as the positive control. The data represent the mean values of three experiments ± SE.**P* < 0.05, ***P* < 0.01, ****p* < 0.001 compared to co-culture with hy926 cells.
